# Targeting PP2A for cancer therapeutic modulation

**DOI:** 10.20892/j.issn.2095-3941.2022.0330

**Published:** 2022-11-01

**Authors:** Halle Ronk, Jared S. Rosenblum, Timothy Kung, Zhengping Zhuang

**Affiliations:** 1 Neuro-Oncology Branch, National Cancer Institute, National Institutes of Health, Bethesda, MD 20892, USA

**Keywords:** Chemo-sensitization, clinical trials, colorectal cancer, glioblastoma, immunotherapy, LB100, protein phosphatase 2A, PP2A inhibition, radio-sensitization, small molecule inhibitor

## Abstract

Protein phosphatases play essential roles as negative regulators of kinases and signaling cascades involved in cytoskeletal organization. Protein phosphatase 2A (PP2A) is highly conserved and is the predominant serine/threonine phosphatase in the nervous system, constituting more than 70% of all neuronal phosphatases. PP2A is involved in diverse regulatory functions, including cell cycle progression, apoptosis, and DNA repair. Although PP2A has historically been identified as a tumor suppressor, inhibition of PP2A has paradoxically demonstrated potential as a therapeutic target for various cancers. LB100, a water-soluble, small-molecule competitive inhibitor of PP2A, has shown particular promise as a chemo- and radio-sensitizing agent. Preclinical success has led to a profusion of clinical trials on LB100 adjuvant therapies, including a phase I trial in extensive-stage small-cell lung cancer, a phase I/II trial in myelodysplastic syndrome, a phase II trial in recurrent glioblastoma, and a completed phase I trial assessing the safety of LB100 and docetaxel in various relapsed solid tumors. Herein, we review the development of LB100, the role of PP2A in cancer biology, and recent advances in targeting PP2A inhibition in immunotherapy.

## Introduction

In 1949, nitrogen mustard (mechlorethamine) became the first chemotherapeutic agent approved by the U.S. Food & Drug Administration, which was rapidly followed by a wave of chemotherapy development^1^. Classic anti-cancer strategies aimed at actively killing cancer cells include alkylating agents that generate crosslinks in DNA and trigger repair mechanisms to induce apoptosis, antimetabolites that severely disrupt nucleic acid synthesis, and alkaloids that interfere with microtubule polymerization/depolymerization and halt mitosis^2^.

Continued investigations have since revealed mutations in canonical signaling pathways that frequently drive tumorigenesis, such as the Hippo pathway or Wnt/β-catenin signaling^3^. Targeting these pathways *via* small molecule inhibitors or monoclonal antibodies has been a focus of more contemporary approaches to cancer treatment. Combining some of these neoadjuvant approaches with standard-of-care chemotherapy or radiotherapy appears to “sensitize” tumor cells to treatment. Negative regulation of protein phosphatase 2A (PP2A) as an adjuvant therapy has demonstrated such chemo- and radio-sensitizing effects^4^.

**Figure 1:**
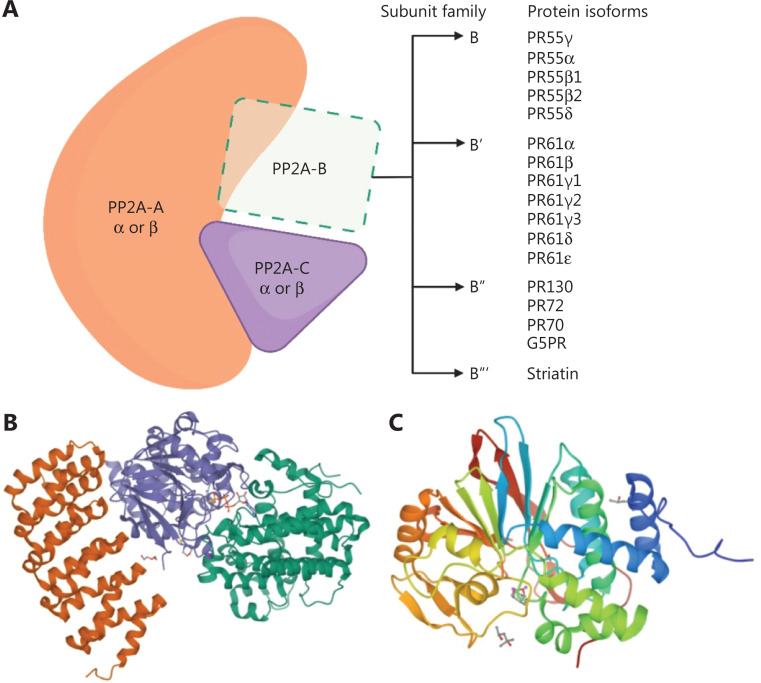
PP2A holoenzyme. (A) Schematic representation of the PP2A holoenzyme, which consists of 3 subunits: a core dimer scaffold A subunit, a regulatory B subunit, and a catalytic C subunit. The scaffold A and catalytic C subunits are encoded by 2 distinct genes, α and β. The regulatory B subunits are classified into 4 unrelated families, each encoded by multiple genes: B (PR55), B′ (PR56/61), B″ (PR48/59/72/130), and B’″ (PR93/110 or striatin). Genetic information was obtained from the UniProt protein sequence database [https://www.uniprot.org/ (accessed on May 1 2022)]. (B) Crystal structure of PP2A bound to PP2A phosphatase activator and ATPγS (PDB 4LAC)^10^, with PP2A-A depicted in orange, PP2A-B depicted in green, and PP2A-C depicted in purple. (C) Crystal structure of PP5 bound to LB100, showing LB100 coordinating with metal ions and key residues at the PP5 catalytic site (PDB 5WG8)^11^. PP2A-C and PP5-C share a common catalytic mechanism^11^. Thus, the interaction between PPP5 and LB100 may provide insight into the interaction between PP2A and LB100.

PP2A, a serine/threonine phosphatase composed of 3 distinct subunits—65-kDa PP2A-A (scaffold subunit), 55-kDa PP2A-B (regulatory subunit), and 37-kDa PP2A-C (catalytic subunit)—constitutes 0.2%–1.0% of the total protein content in mammalian cells and has been implicated in the regulation of diverse cellular processes, including signal transduction, cell cycle progression, DNA replication, gene transcription, and protein translation^5–8^. Whereas the PP2A-A and -C subunits exist in only 2 isoforms, the B regulatory subunit exists in many more^9^. This isoform diversity enables a multitude of holoenzyme assembly combinations, thus partially explaining the diverse cellular functions of PP2A (**Figure 1**).

Initial attempts at targeting PP2A used cantharidin, a naturally occurring compound secreted by blister beetles of the genus *Epicauta chinensis*^12^. Cantharidin has been used in traditional Chinese medicine for centuries, and is associated with bone marrow suppression and urinary tract toxicity^13^. Norcantharidin, the demethylated analogue of cantharidin, was first extracted from the Spanish fly *Mylabris* and was later synthesized in a laboratory setting^14,15^. Further modifications of norcantharidin led to the production of LB100, a synthetic, water-soluble, small-molecule PP2A competitive inhibitor^4^. Since its invention, LB100 has seen broad applications in cancer treatments. Preclinical success has led to new clinical trials of LB100 adjuvant therapies, including a phase I trial in extensive-stage small cell lung cancer (SCLC) [National Library of Medicine (NLM), NCT04560972], a phase I/II trial in myelodysplastic syndrome (NLM, NCT03886662), a phase II trial in recurrent glioblastoma (NLM, NCT03027388), and a completed phase I trial in various relapsed solid tumors (NLM, NCT01837667)^16–19^.

Previous reviews have examined LB100 adjuvant therapies in adult and pediatric nervous system tumors, including glioblastoma, pheochromocytoma, medulloblastoma, diffuse intrinsic pontine glioma, and neuroblastoma^20^. Others have focused on LB100 treatment in systemic solid tumors, including breast cancer, ovarian carcinoma, hepatocellular carcinoma, pancreatic cancer, and sarcoma^4^. Here, we provide an update on LB100 translational studies and insights gained from recent bench-side investigations of LB100 as a modulator of immunotherapy (**Table 1**).

**Table 1:** Overview of reviewed preclinical studies on LB100. Studies reflect a review of the literature as of May 1, 2022.

Investigators (year)	Tumor type	Treatment method	Outcome
Cui et al.^21^ (2020)	Glioblastoma	LB100 and CAR-T cell therapy	Anti-CAIX CAR-T cell therapy and LB100 combination therapy significantly increased tumor-infiltrating lymphocytes (*P* < 0.05) and prolonged survival (*P* < 0.001) in xenograft U251-Luc GBM mice.
Maggio et al.^22^ (2020)	Glioblastoma	LB100 and PD-1 inhibition	Combination therapy, compared with monotherapy, significantly increased survival of syngeneic GL261-Luc GBM mice (*P* < 0.05). Complete tumor regression occurred in 25% of combination-treated mice but was not observed in other treatment or control groups.
Mirzapoiazova et al.^23^ (2022)	Small-cell lung cancer	LB100, carboplatin, atezolizumab, and PD-L1 inhibition	LB100 and carboplatin combination treatment resulted in significantly smaller tumor size in xenograft SCLC mice than controls (*P* < 0.001). LB100 administration also increased carboplatin uptake in tumor cells (*P* < 0.001). Triple therapy with LB100, the PD-1 inhibitor atezolizumab, and anti-PD-L1 starkly destroyed H446 SCLC tumor cell spheroids, increased infiltration by activated T cells, and increased tumor cell death *in vitro*.
Uddin et al.^24^ (2020)	Triple-negative breast cancer	LB100 only	LB100 monotherapy significantly decreased tumor volume in mice bearing MB468 TNBC xenografts.
Yen et al.^25^ (2021)	Colorectal cancer, adenocarcinoma, triple-negative breast cancer, and pancreatic cancer	LB100 and PD-1 inhibition	LB100 and anti-PD-1 co-treatment in multiple cancerous syngeneic mouse models resulted in MLH1 protein loss, greater microsatellite instability, and significantly smaller tumor volumes than those in control groups (*P* = 0.01), corresponding to increased tumor neoantigen expression.
Zhang et al.^26^ (2015)	Osteosarcoma	LB100 and cisplatin	Combination therapy, compared with cisplatin or LB100 alone, resulted in significantly smaller tumor volumes in mice bearing 143B OS xenografts (*P* < 0.05). Pulmonary metastases were observed in only 20% of combination-treated mice, compared with 80% of mice treated with cisplatin alone mice and 100% of mice treated with LB100 alone.
Liu et al.^27^ (2018)	Mucoepidermoid carcinoma	LB100 and cisplatin	Co-treatment of MEC cells with LB100 and cisplatin decreased PP2A activity to 84.98% of control levels *in vitro*. Combination-treated xenograft UM-HMC1 MEC mice also exhibited a significantly smaller tumor volume than did mice treated with either drug alone.
Song et al.^28^ (2021)	Esophageal squamous cell carcinoma	LB100 and paclitaxel	LB100 administration attenuated PP2A expression and decreased MCL1 protein levels (*P* < 0.001) in paclitaxel-resistant ESCC *in vitro*. *In vivo*, mouse models of DR150 paclitaxel-resistant ESCC were treated with LB100 monotherapy and showed significant inhibition of tumor growth (*P* < 0.05).
Hu et al.^29^ (2017)	Acute myelogenous leukemia	LB100 and daunorubicin	LB100 monotherapy increased the proportion of patient-derived AML cells in G2/M phase from 13.4% to 31.5% *in vitro*. LB100 and daunorubicin combination therapy significantly increased cytolysis in these cell lines *in vitro* (*P* < 0.01).
Lai et al.^30^ (2018)	Chronic myelogenous leukemia	LB100 and dasatinib	Combination therapy resulted in significantly fewer BCR-ABL transcripts in transgenic CML mice than controls (*P* < 0.01). The combination also yielded a survival benefit over dasatinib monotherapy (*P* = 0.018) and vehicle (*P* = 0.001).
Ho et al.^31^ (2018)	Meningioma	LB100 and radiation	LB100 and concomitant radiation induced cell death by mitotic catastrophe *in vitro*. Mice bearing malignant IOMM-LEE meningioma xenografts demonstrated a survival benefit when treated with combination therapy compared with either monotherapy (*P* < 0.05).
Hao et al.^32^ (2018)	Chordoma	LB100 and radiation	Co-treatment increased the proportion of cells arrested in G2/M phase of the cell cycle *in vitro*. Tumor size was significantly smaller in xenograft U-CH1 chordoma mice treated with combination therapy than in mice in the control (*P* = 0.028), LB100 alone (*P* = 0.0014), or radiation alone (*P* = 0.0273) groups.

## LB100: a modulator of immunotherapy

### Glioblastoma

Glioblastoma (GBM), a malignant tumor derived from glial cells or their precursors, is the most common primary brain tumor among adults^33^. GBM is characterized by highly aggressive behavior and poor prognosis, with a median overall survival of 15–23 months and a 5-year survival rate below 6%^33^. Standard-of-care treatment involves maximal safe surgical resection, radiation therapy, and concomitant temozolomide (TMZ) treatment^34^. Despite rigorous research and exploration of new chemotherapeutic agents, minimal progress has been made in extending life expectancy or decreasing the tumor recurrence rate among patients with GBM.

Immunotherapy has recently emerged as a potential new treatment for GBM^35^. The blood-brain barrier (BBB) was previously thought to severely limit the entry of immune cells into the central nervous system (CNS), thus delaying the application of immunotherapy to CNS tumors^36^. This dogma has since been challenged by an improved understanding of immune cell trafficking in the CNS gained through findings indicating that T cells can effectively penetrate the BBB, and that the BBB is often broken down in brain tumors, thus increasing permeability to immune cells^37–40^. As a result of these new data, interest in immunotherapy for CNS tumors has been reinvigorated, primarily in 2 major fronts: chimeric antigen receptor (CAR)-engineered T cells and immune checkpoint inhibition.

Several challenges preclude the successful use of CAR-T cells, which are currently designed against specific tumor neoantigens and are injected either centrally or peripherally for trafficking to CNS tumors in the treatment of GBM. These challenges include 1) the limited penetration of CAR-T cells into solid tumors and 2) their suboptimal activation within immunosuppressive tumor microenvironments^41^. PP2A has been identified as an independent negative regulator of CD8^+^ cytotoxic T cell effector function^42^. Because CAR-T cells are CD8^+^ T cells with engineered antigen specificity, PP2A modulation can be exploited in CAR-T therapy with the goal of improving CAR-T efficacy. To this end, Cui et al.^21^ combined LB100 with CAR-T therapy against carbonic anhydrase IX (CAIX) in mice carrying U251-Luc glioma xenografts. The combination significantly increased tumor-infiltrating lymphocytes (*P* < 0.05) and prolonged mouse survival compared to untreated controls (*P* < 0.001)^21^. These results suggest that LB100 may be a helpful adjuvant for immunotherapy by augmenting cytotoxic T cell function and recruiting host immune cells to tumors.

Other studies have combined LB100 with immune checkpoint inhibitors. Programmed death-1 (PD-1), a class of receptors expressed on T cell surfaces, plays an important role in maintaining immune tolerance to self-antigens^43^. Tumor cells aberrantly expressing PD-1 ligand (PD-L1) can prevent T cell activation and escape the host immune system^43^. When used as monotherapy for GBM, PD-1 blockade has not shown clinical benefit^44^. This failure may be partly attributable to the low basal PD-L1 expression in tumors, given that less than 3% of GBM cells express PD-L1^45^. However, Maggio et al.^22^ reported significantly greater survival in syngeneic mice bearing GL261-Luc tumors receiving combination LB100 and PD-1 treatment compared with monotherapy alone (*P* < 0.05). Further, tumors regressed completely in 25% of mice receiving combination therapy but not in mice in either monotherapy group^22^. These studies suggest that LB100 may aid in extending immunotherapy to GBM management *via* immune checkpoint modulation.

### SCLC

SCLC constitutes approximately 15% of all lung cancer diagnoses and is characterized by a high proliferative rate, early metastasis, and poor prognosis^46^. Standard-of-care treatment incorporates radiation with platinum-based chemotherapy^46^. Mirzapoiazova et al.^23^ combined LB100 and carboplatin treatment in mice with SCLC subcutaneous xenografts and observed significantly smaller tumor sizes than those in the vehicle control group (*P* < 0.001). Although combination therapy, compared with carboplatin alone, provides only a modest tumor size reduction, it has been found to increase carboplatin uptake into tumor cells (*P* < 0.001)^23^. Later studies examined the efficacy of concomitant treatment with LB100, the PD-1 checkpoint inhibitor atezolizumab, and a PD-L1 small molecule inhibitor *in vitro*^23^. In H446 SCLC spheroids plated in the presence of T cells and treated with this triple therapy, destruction of the tumor spheroids, infiltration by activated T cells, and increased tumor cell cytotoxicity have been observed^23^. These encouraging results suggest that LB100 may have a role as an adjuvant for SCLC treatment that enhances host immune cell anti-tumor activity.

### Triple-negative breast cancer (TNBC)

TNBC is a subset of breast cancer that lacks the classic progesterone receptors, estrogen receptors, and HER2 amplification, which are targeted by common endocrine therapies^47^. Consequently, TBNC is associated with poorer outcomes, earlier relapse, and poor response to conventional therapy, yet with high susceptibility to tumor necrosis factor-related apoptosis-inducing ligand (TRAIL)-induced apoptosis^47,48^. TRAIL selectively induces the extrinsic apoptotic pathway, resulting in the downstream activation of procaspase-8 and the executioner caspases -3, -6, and -7^49^. PP2A promotes TRAIL resistance by attenuating the phosphorylation and activation of procaspase-8^50^. Therefore, inhibition of PP2A may overcome TRAIL resistance and promote tumor cell death. Supporting this hypothesis, Uddin et al.^24^ demonstrated that LB100 monotherapy results in significantly lower tumor volume in mice bearing MB468 TNBC tumor xenografts than in untreated controls. These results suggest that LB100 may serve as a novel therapy for TNBC by overcoming PP2A-associated apoptotic resistance.

### Colorectal cancer (CRC)

CRC arises from neoplastic epithelial cells of the large intestine and is responsible for approximately 2 million new cases and 1 million deaths reported annually worldwide^51^. Local resection is the mainstay of treatment, but extracolonic metastases occur in approximately 25% of cases and require systemic chemotherapy^51^. Given the rapid resistance to chemotherapy, new therapeutic targets are needed to improve patient outcomes.

One potential target is the DNA mismatch repair (MMR) pathway. Defective MMR occurs in approximately 15% of sporadic CRC cases, and patients with defective MMR have a more favorable prognosis than those without defective MMR^52^. Defective MMR is typically associated with mutations or epigenetic inactivation in one of the 2 major MMR genes, *MLH1* and *MSH2*^53^. Mutations in either gene lead to high-frequency microsatellite instability (MSI)^54^. Yen et al.^25^ found that deletion of the *Ppp2r1a* gene, which encodes PP2A-A, in murine Lgr5^+^ intestinal crypt cells *in vitro* leads to MMR-deficient genetic signatures and positive MSI assay results. These findings have been corroborated in humans: CRC data from The Cancer Genome Atlas Project has revealed that MSI is associated with diminished mRNA and protein levels of PPP2R1A^25^. These data suggest that PP2A plays a role in maintaining normal MMR, and aberrant regulation of PP2A is associated with MSI. The induction of MSI in CRC leads to (1) more frequent genetic errors during active transcription and (2) translation of abnormal proteins, called neoantigens, which can be targeted by classic anti-cancer therapies^52^. This targetability is clinically meaningful because MSI CRC has a more favorable prognosis than non-MSI CRC^52^.

To clarify the mechanism triggering MSI, Yen et al.^25^ examined the histology of mice bearing syngeneic CT26 CRC tumors and found that PP2A-deficient mice show more tumor-infiltrating cytotoxic T cells than PP2A-wildtype mice. They hypothesized that *Ppp2r1a* knockdown converts microsatellite stable “cold” tumors that escape immune recognition to MSI “hot” tumors that attract immune cells by increasing tumor neoantigen expression^25^. To this end, the authors identified 270 missense transcripts, corresponding to 220 genes, shared by 3 PP2A-deleted tumor samples but not found in any PP2A-wildtype tumor samples^25,55^. These results suggest that PP2A inhibition increases genetic errors in cancer cells *via* MSI, increased tumor neoantigens, and subsequent cytotoxic T cell attraction in CRC^25^. Further, PP2A inhibition specifically enhances the immune response against tumor neoantigens to the point of overcoming the checkpoint inhibition commonly used by tumor cells to escape the host immune system^25^. Overall, the changes caused by PP2A inhibition promote MSI in CRC, thus improving targeting against tumors.

Later studies examined the response to LB100 in mice carrying syngeneic CT26 colorectal cancer, MC38 adenocarcinoma, 4T1 triple-negative breast cancer, and Pan18 pancreatic cancer^25^. After co-treatment with LB100 and anti-PD-1, these syngeneic mice demonstrate MLH1 protein loss, greater MSI frequency, and significantly lower tumor volume than controls (*P* = 0.01)^25^. Together, these data indicate an exciting new role of LB100 as a promoter of neoantigen expression and immune activation in various solid neoplasms.

One recent CRC clinical trial has raised the question of whether combining LB100 and PD-1 blockade might further increase the efficacy of immunotherapy in human CRC. This phase II clinical trial used single agent dostarlimab, an anti-PD-1 monoclonal antibody, in patients with MMR-deficient stage II or III rectal adenocarcinoma (NCT, NCT04165772)^56,57^. Remarkably, all 12 study participants demonstrated complete tumor regression at 6 months^57^. Future clinical trials might combine LB100 and anti-PD-1, given the success achieved in preclinical models^25^.

## Updates: LB100 as a chemo-sensitizer

### Osteosarcoma (OS)

OS is the most common primary malignant bone tumor and the third most common cancer in adolescence^58^. OS treatment comprises maximal tumor resection and a standard chemotherapy cocktail incorporating methotrexate, doxorubicin, cisplatin, and ifosfamide^58^. Even when maximal surgical resection is achieved, approximately 80% of patients have been found to develop metastases, thus spurring a search for new chemo-agents that can effectively target metastatic OS^59^. In mice with 143B OS tumor xenografts, Zhang et al.^26^ have shown that LB100 and cisplatin combination therapy, compared with cisplatin or LB100 alone, greatly decreases OS tumor growth (*P* < 0.05). Further, only 20% of mice in the combination cohort developed pulmonary metastases as compared with 80% in the cisplatin alone group and 100% in the LB100 alone group^26^. Histologic analysis indicated that combination therapy increased levels of phosphorylated checkpoint kinase-1 (Chk1) and decreased levels of phosphorylated p53^26^. LB100 is likely to attenuate p53 activation and promote Chk1-mediated G1/S arrest in response to chemotherapy-induced DNA damage, thus facilitating inappropriate entry into mitosis and causing cell death through mitotic catastrophe.

### Mucoepidermoid carcinoma (MEC)

MEC is a rare malignancy that arises from the salivary glands^27^. The treatment for high-grade MEC involves surgical resection, although radiotherapy and chemotherapy are used intermittently^27^. Interestingly, preclinical models have shown that some chemo-reagents can increase PP2A activity, a response associated with MEC tumor progression^60^. For example, Liu et al.^27^ have shown that MEC cells treated with cisplatin *in vitro*, compared with untreated tumor cells, exhibit greater PP2A activity (116.7% that of control), as measured by phosphatase detection assays, whereas co-treatment with LB100 and cisplatin decreases PP2A activity to 85% of control levels^27^. This decrease in PP2A activity is clinically significant, because it has been found to correlate with diminished tumor burden in *in vivo*^27^. Interestingly, because decreasing PP2A below ∼70% of wild-type activity causes dramatic cytoskeletal changes and apoptosis in healthy and tumor cells alike, eliciting a further decrease would not be an appropriate therapeutic target^61^. Mice carrying subcutaneous UM-HMC1 MEC xenografts showed significantly lower tumor volume after 28 days of combination therapy than either monotherapy (*P* < 0.05)^27^. Thus, elevated PP2A levels in MEC may be associated with higher tumor burden, but adjuvant LB100 therapy reverses this trend.

### Esophageal squamous cell carcinoma (ESCC)

ESCC, the sixth leading cause of cancer death worldwide, is an aggressive malignancy arising from the esophagus^62^. Cytotoxic chemotherapies such as paclitaxel are mainstays of treatment^63^. Paclitaxel binds the β-subunit of tubulin, thus preventing microtubule depolymerization and inducing arrest at G2/M phase^64^. Despite the success of paclitaxel, eventual resistance is inevitable. Song et al.^28^ observed that resistance to a nanoparticle-bound paclitaxel (nab-PTX) developed in 3 ESCC tumor lines in vitro and was associated with greater PP2A expression and stabilization of the anti-apoptotic protein MCL1. Further, this study showed that LB100 treatment attenuated PP2A expression and decreased MCL1 protein levels (*P* < 0.001)^28^. *In vivo*, mice bearing nab-PTX naive KYSE150 ESCC tumor cells have been found to be unaffected by LB100 monotherapy, whereas mice carrying nab-PTX resistant DR150 ESCC tumor cells display significant tumor growth inhibition after LB100 administration (*P* < 0.05)^28^. Thus, LB100 may be used to overcome tumor cell resistance to anti-microtubule agents.

### Acute myelogenous leukemia (AML)

Leukemias are a diverse group of hematologic malignancies that arise from abnormal lymphocytes. AML accounts for 80% of adult acute leukemia cases^65^. Treatment involves co-administration of an anthracycline plus a cytarabine, followed by allogeneic stem cell transplantation^66^. LB100 has shown promise as a monotherapy for AML in preclinical models. *In vitro*, Hu et al.^29^ observed that LB100 monotherapy significantly increased the proportion of SKM-1 AML cells in G2/M phase from 13.4% to 31.5%, thus indicating an increased number of cells arrested in the radio- and chemo-sensitive mitotic phase of the cell cycle. Such mitotic catastrophe represents a form of apoptosis. The potential of LB100 to function as a chemosensitizer for the anthracycline daunorubicin has also been investigated. Compared with monotherapy, the combination has been found to significantly enhance cytolysis in SKM-1 AML cells and 3 primary AML patient samples *in vitro* (*P* < 0.01)^29^. The underlying mechanism is likely to be associated with LB100-induced upregulation of the microRNA miR-181b-1, thereby resulting in downregulation of the anti-apoptotic factor Bcl-2^29^. Thus, treatment of AML cells with LB100 promotes mitotic catastrophe and apoptosis.

### Chronic myelogenous leukemia (CML)

CML is defined by the presence of the Philadelphia chromosome (*BCR-ABL1* fusion gene)^67^. CML treatment is based on tyrosine kinase inhibitors, which antagonize the constitutively active BCR-ABL tyrosine kinase produced by the Philadelphia chromosome^67^. A combination of LB100 and the tyrosine kinase inhibitor dasatinib in a transgenic mouse model of CML has been found by Lai et al.^30^ to result in significantly fewer BCR-ABL transcripts than those in control mice (*P* < 0.01). Further, combination therapy has been found to confer a survival benefit over dasatinib monotherapy (*P* = 0.018) and vehicle-treated mice (*P* = 0.001)^30^.

One hypothesis potentially explaining CML relapse is persistence of leukemic stem cells, which are maintained by BCR-ABL-associated signaling pathways, such as the Wnt/β-catenin pathway^67^. Mechanistic investigations have suggested that PP2A inhibition plays a role in CML stem cell renewal by modulating Abelson helper integration site-1 (AHI-1)^30^. AHI-1 directly interacts with PP2A and β-catenin, and mediates their interaction^67^. In the presence of functional PP2A, β-catenin is dephosphorylated and spared from ubiquitin-proteasome-mediated degradation^68^. When PP2A is inhibited, β-catenin does not associate with AHI-1 and remains phosphorylated^30^. These events lead to the degradation of β-catenin and subsequent inhibition of downstream signaling^30^. Thus, PP2A inhibition disrupts AHI-1-mediated signaling pathways and consequently allows LB100 to target cancer stem cells in CML.

## Updates: LB100 as a radio-sensitizer

### Meningioma

Meningiomas represent more than 36% of all primary CNS tumor diagnoses and arise from the neural crest belonging to the bony dura, leptomeninges, or brain surface^33,69^. Treatment involves surgical resection or radiation for non-resectable tumors^70^. Ho et al.^31^ investigated concurrent LB100 and radiotherapy in 3 atypical and anaplastic meningioma cell lines *in vitro*: IOMM-LEE, GAR, and CH-157. Combination therapy has been found to induce cell death by mitotic catastrophe and to trigger cell-cycle arrest in G2/M phase as a result of persistent unrepaired DNA damage^31^. Further, IOMM-LEE-bearing mice demonstrate a survival benefit when treated with combination therapy *vs.* radiation alone or LB100 monotherapy (*P* < 0.05)^31^. These results illustrate a novel role of LB100 chemotherapy for meningiomas.

### Chordoma

Chordomas are rare, aggressive, locally invasive malignant bone tumors that are remnants of the embryologic notochord and arise within the axial skeleton^71^. Treatment consists of *en bloc* excision with postoperative radiotherapy^71^. Treatment of U-CH1 chordoma cells with LB100 and radiation dual therapy *in vitro* has been found by Hao et al.^32^ to lead to more cells in the radiation-sensitive G2/M phase of the cell cycle. In mice bearing U-CH1 subcutaneous xenografts, combination therapy has been found to severely attenuate tumor growth with compared to the control group (*P* = 0.028), LB100 monotherapy (*P* = 0.0014), and radiation alone (*P* = 0.0273) groups^32^. Histology performed on these mice revealed the presence of aberrant multinucleated tumor cells after LB100 treatment, suggesting that PP2A is essential for mitosis^32^. Thus, LB100 promotes chordoma susceptibility to radiotherapy by inducing mitotic catastrophe.

## Discussion

Protein kinases and phosphatases are key targets in the development of modern anti-cancer therapies, yet their success has been tempered by toxicities affecting the heart, lungs, liver, kidneys, thyroid, skin, blood coagulation, gastrointestinal tract, and nervous system^72,73^. Okadaic acid, a lipophilic marine toxin produced by several species of phytoplankton inhibits multiple protein phosphatases (PPs)—PP1, PP2A, PP2B, PP4, and PP5—and is associated with various systemic toxicities including cytotoxicity through apoptosis induction, neurotoxicity associated with tau protein hyperphosphorylation, immunotoxicity *via* modifications on interleukin-1 production, embryotoxicity, and even tumor promotion^74^.

The broad adverse effect profile of nonselective PP inhibitors has encouraged the development of highly selective PP inhibitors in the hope that more selective agents may have less systemic toxicity but retain strong antitumor activity^11^. In contrast to its aforementioned historical predecessors, LB100 is a selective PP inhibitor: it is a well-known PP2A inhibitor, and new evidence suggests that it may also inhibit PP5 *via* the catalytic domain (**Figure 1**)^11^. PP2A-C and PP5-C share a common catalytic mechanism, as evidenced by the high structural similarity of catalytic pockets of both of these enzymes^11^. Interestingly, LB100 has modest selectivity (∼4-7 fold) for PP2A-C *vs.* PP5-C, thus reaffirming LB100 as a selective PP inhibitor^11^.

**Figure 2:**
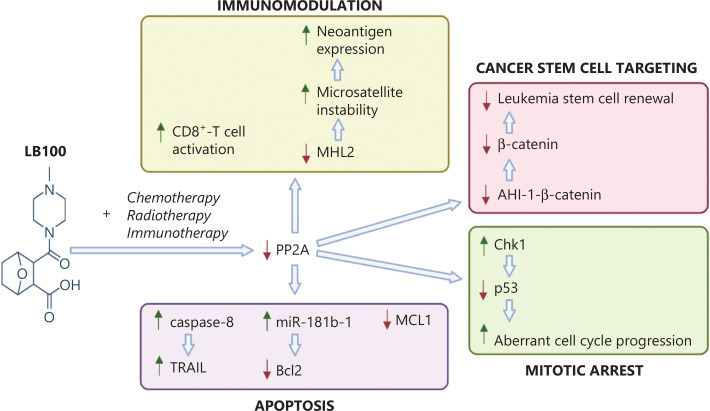
Downstream molecular events after LB100 adjuvant therapy. The schematic diagram summarizes the major downstream molecular events occurring after LB100 is administered concurrently with chemotherapy, radiation, and/or immunotherapy. Increased or decreased expression of the target proteins is indicated by a red or green arrow, respectively. The major effects of LB100 include activating the host immune system, targeting cancer stem cells through canonical signaling pathways, triggering mitotic catastrophe, and promoting tumor cell death through apoptosis.

The mechanisms of action of LB100 against cancer include activating the host immune system, recruiting cytotoxic T cells to tumors, disrupting canonical signaling pathways that maintain cancer stem cells, promoting aberrant regulation of the cell cycle, and preventing tumor cell resistance to apoptosis (**Figure 2**). Perhaps the most striking effect of LB100 is its induction of dramatic cytoskeletal changes. Alterations in the phosphorylation state of cytoskeletal proteins in response to extracellular signals occur *via* changes in the relative activities of protein kinases and phosphatases^75^. PP2A is a counter-regulator of cytoskeletal kinases: it dephosphorylates cytosolic tau and MAP2, initiates microtubule depolymerization, and stabilizes filamentous actin branching, thereby promoting neurite outgrowth and dendritic cell branching^75^. Interestingly, PP2A may also influence the activation of CDK5, a non-cell cycle kinase involved in cytoskeletal regulation^61,76^. However, further investigation is needed to better characterize this relationship.

Clinical studies have reaffirmed LB100 as an effective yet well-tolerated antitumor agent. A completed phase I trial on LB100 in patients with relapsed solid tumors has yielded promising results^77^. Of the 29 patients enrolled, grade 3 adverse events occurred in only 6 (20.7%) patients, whereas 10 (50%) patients achieved stable disease^77^. Adverse reactions included anemia, lymphopenia, decreased creatinine clearance, hyponatremia, and dyspnea^77^. Targeting PP2A inhibition by LB100 is thus a promising new avenue for the development of neoadjuvant cancer treatments. Currently, 3 ongoing clinical trials are investigating the utility of LB100 as an adjuvant therapy or second-line treatment for various solid and hematologic malignancies.
